# A new real-time PCR protocol for detection of avian haemosporidians

**DOI:** 10.1186/s13071-015-0993-0

**Published:** 2015-07-19

**Authors:** Jeffrey A. Bell, Jason D. Weckstein, Alan Fecchio, Vasyl V. Tkach

**Affiliations:** Department of Biology, University of North Dakota, 10 Cornell Street STOP 9019, Grand Forks, ND 58202 USA; Department of Ornithology and Department of Biodiversity, Earth, and Environmental Sciences, Academy of Natural Sciences of Drexel University, 1900 Benjamin Franklin Parkway, Philadelphia, PA 19103 USA

**Keywords:** Avian haemosporidians, *Plasmodium*, *Haemoproteus*, *Leucocytozoon*, Real-time PCR

## Abstract

**Background:**

Birds possess the most diverse assemblage of haemosporidian parasites; including three genera, *Plasmodium*, *Haemoproteus*, and *Leucocytozoon*. Currently there are over 200 morphologically identified avian haemosporidian species, although true species richness is unknown due to great genetic diversity and insufficient sampling in highly diverse regions. Studies aimed at surveying haemosporidian diversity involve collecting and screening samples from hundreds to thousands of individuals. Currently, screening relies on microscopy and/or single or nested standard PCR. Although effective, these methods are time and resource consuming, and in the case of microscopy require substantial expertise. Here we report a newly developed real-time PCR protocol designed to quickly and reliably detect all three genera of avian haemosporidians in a single biochemical reaction.

**Methods:**

Using available DNA sequences from avian haemosporidians we designed primers R330F and R480RL, which flank a 182 base pair fragment of mitochondrial conserved rDNA. These primers were initially tested using real-time PCR on samples from Malawi, Africa, previously screened for avian haemosporidians using traditional nested PCR. Our real time protocol was further tested on 94 samples from the Cerrado biome of Brazil, previously screened using a single PCR assay for haemosporidian parasites. These samples were also amplified using modified nested PCR protocols, allowing for comparisons between the three different screening methods (single PCR, nested PCR, real-time PCR).

**Results:**

The real-time PCR protocol successfully identified all three genera of avian haemosporidians from both single and mixed infections previously detected from Malawi. There was no significant difference between the three different screening protocols used for the 94 samples from the Brazilian Cerrado (*χ*^2^ = 0.3429, df = 2, P = 0.842). After proving effective, the real-time protocol was used to screen 2113 Brazilian samples, identifying 693 positive samples.

**Conclusions:**

Our real-time PCR assay proved as effective as two widely used molecular screening techniques, single PCR and nested PCR. However, the real-time protocol has the distinct advantage of detecting all three genera in a single reaction, which significantly increases efficiency by greatly decreasing screening time and cost. Our real-time PCR protocol is therefore a valuable tool in the quickly expanding field of avian haemosporidian research.

## Background

Haemosporidians are protozoan parasites that infect vertebrate blood cells and are transmitted by dipteran vectors [1–5]. Haemosporidians are one of the most widely studied groups of vertebrate parasites, because members of the genus *Plasmodium* have severe impacts on human health [6, 7] and their evolutionary history is generally not fully understood [8]. Birds possess the highest diversity of haemosporidian parasites, including three genera, *Plasmodium, Haemoproteus*, and *Leucocytozoon* [4]. Studies of avian haemosporidians have a long history being first described by Danilewsky [9] and later used as a model for human malaria [4, 6, 10]. With the discovery of rodent malaria [11] avian haemosporidians lost their importance as laboratory models. Consequently, they were relegated to the status of a group of limited interest, studied mainly in connection with impacts of these parasites on wild and domestic bird populations [4].

The past two decades have seen a dramatic increase in the study of these parasites as tools to test evolutionary theories of parasite-host interactions [12–18] and the cost of parasitism on host populations [19–24]. The growth in this field is directly tied to the development of a standard nested PCR protocol for amplifying a portion of the haemosporidian cytochrome *b* gene [25–27] and the subsequent development of the MalAvi database of avian haemosporidian lineages [28] (http://mbio-serv2.mbioekol.lu.se/Malavi/). Prior to the development of these resources, the main method to identify these parasites was microscopic examination of blood films, which requires expertise in making, staining, and examining such films. Although examination of blood films is an effective way for identifying and quantifying parasites [29], the expertise needed to screen blood films takes time to develop, and chronically infected birds with low parasitemia can be missed [27, 30]. Although morphological data remain essential to link genetic lineages with known morphospecies [29], molecular identification requires only minimal training, does not require quality blood films, and is generally accepted to be more sensitive than microscopy [30–34]. It is also much faster and allows screening of large numbers of samples in a relatively short time.

The PCR protocols initially developed by Bensch *et al.* [25], and modified by Hellgren *et al.* [26], and Waldenström *et al.* [27] are widely used today. They rely on using two nested PCR amplifications of a 478 bp fragment of the cytochrome *b* gene, one set of nested PCR for *Haemoproteus/Plasmodium* [25, 27] and a separate set for *Leucocytozoon* [26]. Although effective at both screening and amplifying haemosporidian parasite DNA, the time and amount of reagents necessary for running nested reactions can be limiting when screening large numbers of samples. Fallon *et al.* [35] worked around this issue by developing an initial standard PCR screening protocol that amplified a 154 bp fragment of the conserved rDNA region of the mitochondrial genome of *Haemoproteus* and *Plasmodium*, although it did not identify *Leucocytozoon*. Only positive samples from screening were subsequently amplified by regular PCR for cytochrome *b* and sequenced. This increased the speed at which large sets of samples could be screened, but still required the gel electrophoresis of hundreds or thousands of PCR products. Subsequently, researchers who used the Fallon *et al.* [35] protocol for initial screening moved to various nested PCR protocols, e.g.[36, 37], to improve the chances of amplifying haemosporidian DNA from hosts with low intensity of infection.

The use of real-time PCR to screen samples for presence of viral [38–40], bacterial [41–43], or parasite [44–46] DNA has become a useful and common method of determining pathogen prevalence in host populations. Although real-time PCR has been used for avian haemosporidians, it has generally been used to determine level of parasitemia [47–50] or for detecting specific lineages [22, 51–53]. The usefulness of real-time PCR as a large scale screening tool for haemosporidian DNA in avian blood samples has been only minimally explored [54] and never done for all three genera with a single reaction. Here we report the development of a real-time PCR protocol that can identify infections of any of three haemosporidian genera in a single screening reaction using a 182 bp fragment of the conserved RNA region of the mitochondrial genome.

## Methods

**Fig. 1:**
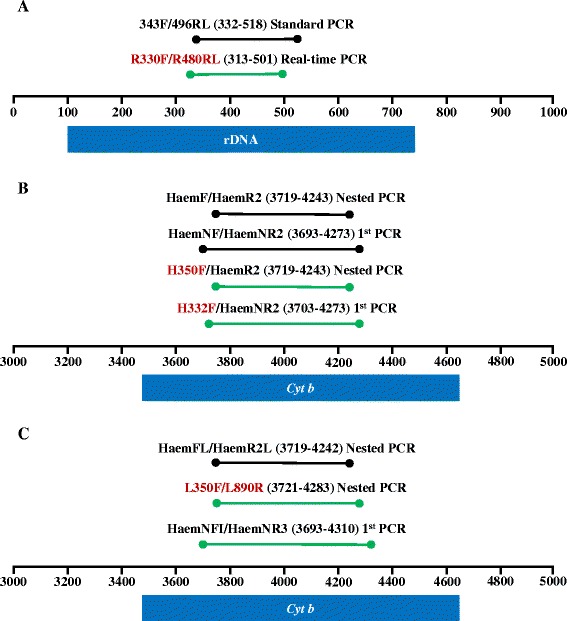
Primer positions of rDNA primers for standard and real-time PCR (**a**) and cytochrome b primers for nested PCR for *Haemoproteus/Plasmodium* (**b**), and *Leucocytozoon* (**c**). Blue bars denote location of the target genes on the mitochondrial genome of *Plasmodium relictum* (NC012426). The spans of amplified DNA fragments are indicated in parentheses behind each primer pair. Fragments in green are those that we recommend for use in avian haemosporidian detection (**a**) and amplification by nested PCR (**b**, **c**). Primers in red represent new primers developed for avian haemosporidians either herein or in [17]

Design of primers that could successfully amplify all three genera in a single real-time reaction required determining a gene region that is more conserved than the standard 478 bp fragment of the cytochrome *b* gene [26, 27]. The conserved rDNA region of the mitochondrial genome was a good target because it is quite conserved in avian haemosporidians and has been previously used to screen for *Haemoproteus* and *Plasmodium* infections [35]. Available avian haemosporidian mitochondrial sequences from GenBank (Table 1) that contained the conserved rDNA region were aligned using BioEdit v7.2.0 [55]. Although the primers described by Fallon *et al.* [35] did not match *Leucocytozoon* sequences, a region adjacent to these primers proved to be sufficiently conserved for detection of all three genera. The forward primer R330F and reverse primer R480RL were designed, flanking a 182 base pair fragment (Fig. 1, Table 2).

**Table 1:** List of GenBank sequences used to design real-time PCR primers to detect haemosporidian rDNA. Accession numbers and the associated haemosporidian species/lineage are given

Accession Number	Haemosporidian species/lineage
FJ168562	*Haemoproteus columbae*
AY733087	*Haemoproteus* sp. jb1. JA27
AB302215	*Leucocytozoon caulleryi*
FJ168564	*Leucocytozoon fringillinarum*
FJ168563	*Leucocytozoon majoris*
NC009336	*Leucocytozoon sabrezesi*
AB250690	*Plasmodium gallinaceum*
AB250415	*Plasmodium juxtanucleare*
KC138226	*Plasmodium lutzi*
NC012426	*Plasmodium relictum*

**Table 2:** Primer sequences for real-time and nested PCR protocols, along with sequence of positive control used for real time PCR reactions. Sequencing primers are also listed

Protocol/primer	Primer sequence
Real-Time PCR – *Haemoproteus, Plasmodium, Leucocytozoon*	
R330F^a^	5′- CGTTCTTAACCCAGCTCACG - 3′
R480RL^a^	5′- GCCTGGAGGTWAYGTCC – 3′
*P. relictum* – Pos. Control	5′- GGGAACAAACTGCCTCAAGACGTTCTTAACCAGCT
(Accession # NC012426)	CACGCATCGCTTCTAACGGTGAACTCTCATTCCAA
	TGGAACCTTGTTCAAGTTCAAATAGATTGGTAAGG
	TATAGCGTTTACTATCGAATGAAACAATGTGTTCC
	ACCGCTAGTGTTTGCTTCTAACATTCCATTGCTTAT
	AACTGTATGGACGTAACCTCCAGGCAAAGAAAAT
	GACCGGTC – 3′
Nested PCR – *Haemoproteus* and *Plasmodium*	
H332F^a^	5′ - GAGAATTATGGAGYGGATGGTG - 3′
HAEMNR2^b^	5′ - AGAGGTGTAGCATATCTATCTAC- 3′
H350F^a^	5′ – GGTGTTTTAGATATATGCATGC - 3′
HAEMR2^c^	5′ - GCATTATCTGGATGTGATAATGGT - 3′
Nested PCR – *Leucocytozoon*	
HAEMNFI^d^	5′ - CATATATTAAGAGAAITATGGAG - 3′
HAEMNR3^d^	5′ - ATAGAAAGATAAGAAATACCATTC - 3′
L350F^e^	5′ - GGTGTTTTAGATACTTA -3′
L890R^e^	5′ - TACAATATGTTGAGGTGTTTG - 3′
Sequencing – *Haemoproteus* and *Plasmodium*	
FIFI^f^	5′ – GGGTCAAATGAGTTTCTGG - 3′
R2^f^	5′ - GCTGTATCATACCCTAAAGG - 3′
Sequencing – *Leucocytozoon*	
L545F^e^	5′ - ACAAATGAGTTTCTGGGGA - 3′
L825R^e^	5′ - GCAATTCCAAATAAACTTTGAA - 3′

^a^Designed for this study

^b^[27]

^c^[25]

^d^[26]

^e^[17]

^f^[56]

These primers were tested using DNA extracted from avian blood stored on Whatman FTA Classic Cards or 95 % ethanol and liver samples stored in 95 % ethanol. DNA was extracted using the Qiagen DNeasy 96 Blood and Tissue kit (Qiagen, Valencia, CA), following the Qiagen dried blood spot protocol for blood stored on Whatman FTA Classic Cards and the Qiagen tissue protocol for both blood and liver stored in 95 % ethanol. Since blood coagulates in 95 % ethanol, sterilized wooden applicators were used to transfer a small portion of the clot representing approximately 2 mm^3^ into each extraction tube. Both liver and coagulated blood samples required overnight incubation at 56 °C for appropriate digestion. Both the American Ornithologist’s Union (http://www.nmnh.si.edu/BIRDNET/guide) and University of North Dakota Animal Care and Use Committee guidelines (Project # 1402-1) for ethically collecting avian blood and tissue samples were strictly followed.

**Fig. 2:**
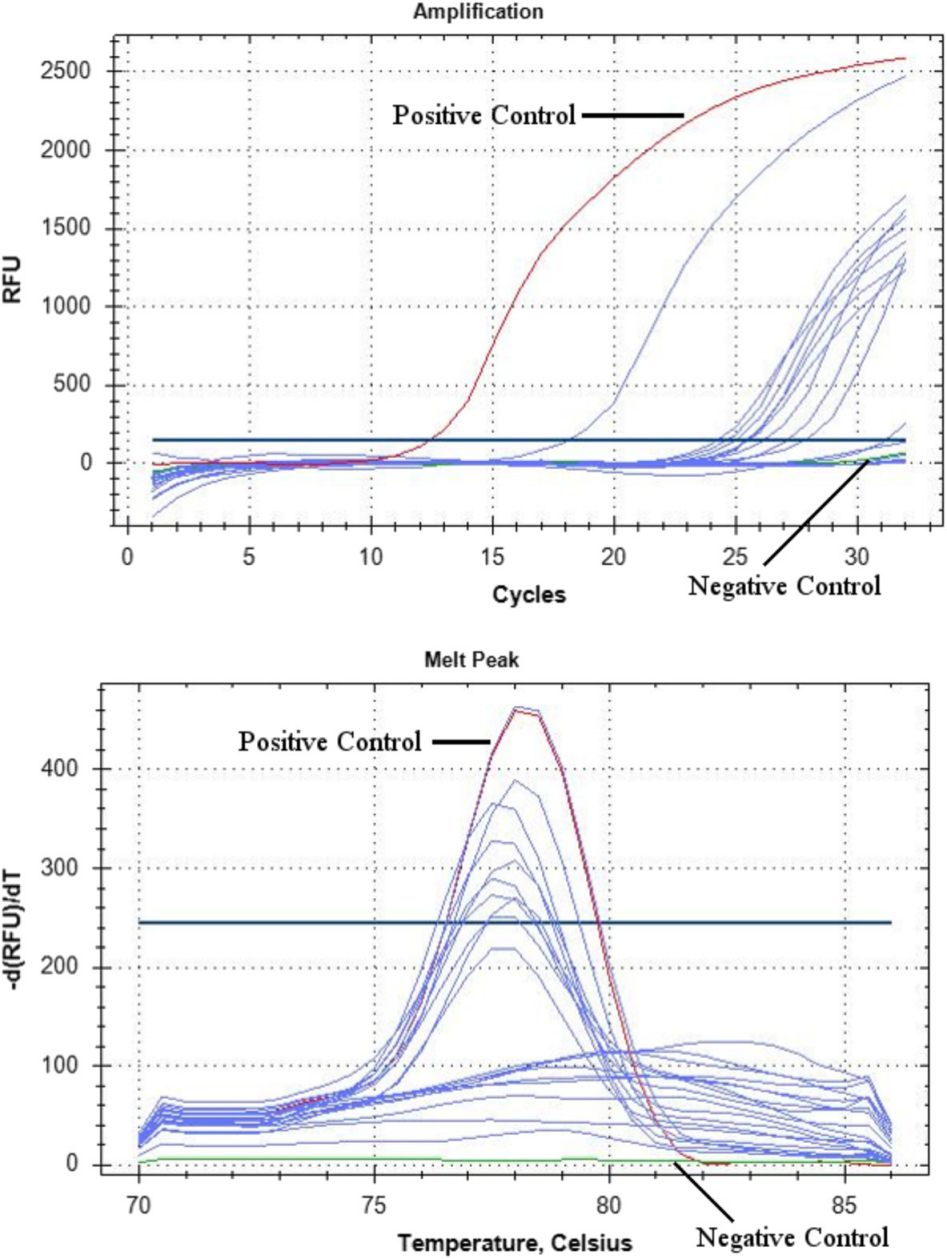
Amplification and melt peak curves from real-time PCR amplification of rDNA from avian blood samples. Positive (*Plasmodium relictum*) control, shown in red, and negative (water) control, shown in green, are indicated in the curves

All reactions were carried out using iTaq universal SYBR Green Supermix on a CFX96 real-time thermocycler (Bio-Rad, Hercules, CA). The total volume of the reactions was 15 μl, with 7.5 μl of SYBR Green Supermix, 0.6 μl of each primer (10 μM concentration), 3.3 μl of molecular grade water, and 3 μl of DNA template (the volume established empirically, approximately 20 ng/μl). The following cycling conditions were used: 95 °C for 30 s, followed by 35 cycles of 95 °C for 30 s and 53 °C for 35 s (with a plate read) followed by a final melt curve analysis using instrument default settings. Positive and negative controls were included in all runs. The positive control used was a synthetic double stranded DNA product (G-Block - IDT DNA, Coralville, IA) designed from a 220 bp fragment of the conserved rDNA region of *Plasmodium relictum* (Accession # NC012426) (Table 1). The positive control of *Plasmodium relictum* produced a melt curve peak at 78.5 °C (Fig. 2).

This protocol was initially tested on samples positive for *Plasmodium*, *Haemoproteus*, or *Leucocytozoon,* samples with mixed infections, and known negative samples, from a previous study of haemosporidians from Malawi, Africa [17]. These samples had been previously screened by nested PCR and microscopy [17] and were from 16 host species, representing 15 genera, 13 families, and 7 orders.

To further test this protocol 94 samples were selected from 791 samples collected from the Cerrado biome of Brazil and previously screened for haemosporidian parasites [36] using the Fallon *et al.* protocol [35]. These samples were obtained from four host species, *Myiarchus swainsoni, Neothraupis fasciata, Nystalus chacuru,* and *Volatinia jacarina*, and were rescreened with the real-time protocol and also amplified using nested PCR protocols (described below) to amplify the cytochrome *b* gene. This not only allowed for testing the effectiveness of the real time protocol, but also enabled comparison between the three different screening methods (single PCR, nested PCR, real-time PCR). Results for these screening methods were analysed using a 2 × 3 chi-square contingency table using the package Rcmdr in program R [56].

Two modified nested PCR protocols were used to amplify fragments of the cytochrome *b* gene (Table 2). The protocol for *Haemoproteus/Plasmodium* was based on the standard protocol of Waldenström *et al.* [27] but with newly designed forward primers, H332F and H350F (Fig. 1, Table 2), which match more closely with available GenBank sequences. The protocol produces a 477 bp fragment, which is only one base pair shorter than the fragment produced by the Waldenström *et al.* protocol [27]. The *Leucocytozoon* protocol uses the initial primer sets described by Hellgren *et al.* [26] but with newly designed nested primers [17] (Fig. 1, Table 2). This new protocol produces a 526 bp fragment that encompasses the 478 bp fragment produced by the Hellgren protocol [17].

All nested PCRs were run using OneTaq Quick-Load 2X Master Mix with standard buffer (New England Biolabs, Ipswich, MA) in 20 μl reactions. The initial PCR amplifications included 10 μl of OneTaq Master Mix, 1 μl of each primer (10 μM concentration), 3 μl of molecular grade water, and 5 μl of template (the volume established empirically, approximately 20 ng/μl). The nested PCR amplifications differed in using 5 μl of water and 3 μl of PCR product as template. The following protocol was used for all reactions; 95 °C for 3 min, then followed by 20 cycles (first amplification)/35 cycles (nested amplification) of 95 °C for 30 s, 50 °C for 45 s, and 68 °C for one minute, followed by a final elongation at 68 °C for 5 min. Negative controls were included in all nested PCR runs. Each sample identified as positive by real-time PCR underwent two separate nested PCR amplifications, one for *Haemoproteus/Plasmodium* using our modified Waldenström protocol and one for *Leucocytozoon* [17].

PCR products were run on 1.25 % agarose gels, stained with ethidium bromide, and visualized under UV light. Positive PCR products were purified using ExoSAP-IT (Affymetrix, Santa Clara, CA) and sequenced using BigDye terminator v3.1 cycle sequencing kit (Applied Bio systems, Foster City, CA). The primers FIFI and R2 [57] were used for sequencing of *Haemoproteus* and *Plasmodium* and the primers L545F and L825R [17] were used for *Leucocytozoon* (Table 1). Sequencing reaction products were precipitated with ethanol, dried by vacuum centrifuge, re-suspended with 10 μl of dH2O, and run on an ABI 3100 DNA sequencer (Applied Bio systems, Foster City, CA). Forward and reverse sequences were visualized and assembled using Sequencher v.5.0.1 (Gene Codes Corp., Ann Arbor, MI). Assembled sequences were aligned using BioEdit v7.2.0 [55] and collapsed to unique haplotypes using the FaBox haplotype collapse and converter tool [58]. Sequence identities were verified with a local BLAST against the MalAvi database [28] using BioEdit v7.2.0 [55].

## Results

The real-time PCR protocol successfully identified all single infections of *Plasmodium*, *Haemoproteus*, and *Leucocytozoon* previously detected by standard nested PCR protocol and microscopy [17] from samples collected in Malawi, Africa. For all three genera the melt peaks generally occurred between 78 to 79 °C, but variability existed, with some lineages producing peaks slightly above or below this range. The assay also detected all samples from the same collection with mixed infections of *Plasmodium/Haemoproteus*, *Plasmodium/Leucocytozoon*, *Haemoproteus/Leucocytozoon*, and *Plasmodium/Haemoproteus/Leucocytozoon*, but due to the use of a single primer set it was generally not possible to discern mixed infections with the real-time PCR assay. The intensity of infection as determined by blood films had no effect on detection by real-time PCR. It successfully detected the presence of haemosporidians in samples with only one infected red blood cell per 100 fields at 1000× magnification.

There was no significant difference between the three different screening protocols used for the 94 samples from Cerrado (*χ*^2^ = 0.3429, df = 2, P = 0.842) (Table 3). The Fallon protocol identified 49 positive samples, the real-time protocol identified 53 positive samples, and our nested PCR protocol for *Haemoproteus/Plasmodium* (Table 2) identified 51 positive samples (Table 3). The samples were also run using the *Leucocytozoon* nested PCR protocol [17] and all were negative. The real-time protocol identified 45 out of 49 samples previously identified by the Fallon protocol and 48 out of 51 samples identified by our nested PCR protocol. Two samples determined to be positive by both the Fallon *et al.* protocol [35] and the real-time protocol were negative by our nested PCR protocol and three samples were only found positive by the real-time protocol. Both the Fallon protocol and the real-time protocol failed to identify three samples screened as positives by our nested PCR protocol (Table 3).

**Table 3:** Results of single, nested, and real-time PCR tests on 94 samples from Cerrado biome of Brazil. Only samples that were positive by at least one screening method are shown, 36 samples were negative by all three methods. Forty-two samples were positive by all three screening methods (bold text), samples with divergent results are shown individually

Sample ID	Single PCR	Nested PCR	Real-time PCR
**Various (n = 42)**	**Positive**	**Positive**	**Positive**
CE0049	Positive		Positive
CE0051		Positive	Positive
CE0053		Positive	Positive
CE0058			Positive
CE0060		Positive	Positive
CE0068	Positive	Positive	
CE0071			Positive
CE0074			Positive
CE0076			Positive
CE0578	Positive		Positive
CE0581		Positive	Positive
CE0592	Positive	Positive	
CE0594		Positive	Positive
CE0595	Positive	Positive	
CE0597	Positive	Positive	
CE0598	Positive		
TOTAL	49	51	53

After all the new and amended protocols were tested, the real-time protocol was used to screen 2113 samples collected from three Brazilian biomes; Amazonia, Caatinga, and Pantanal and representing 332 host species. Of these 2113 samples, 693 were identified as positive by real-time PCR. Of those 693 infected, we successfully amplified cytocrome *b* with nested primers in 532 samples (77 %) and confirmed their identification by sequencing. These infected individuals included single infections of *Plasmodium* and *Haemoproteus* as well as coinfections of two different haemosporidian taxa, including *Haemoproteus/Haemoproteus*, *Haemoproteus/Plasmodium*, and *Plasmodium/Plasmodium*. No *Leucocytozoon* infections have been detected in this sample which is in agreement with previous reports from the region [4, 59, 60].

## Discussion

The real-time protocol presented herein is highly effective at determining the presence of haemosporidian parasites in avian blood and liver samples. It reliably identified all known positive samples from a recently published study of haemosporidians from birds sampled in Malawi [17] and matched the results of two other standard molecular screening methods. The real-time protocol also successfully detected parasites in more than 2100 samples from Brazil. The results of these three screening methods (single PCR, nested PCR, real-time PCR) were not significantly different when used to screen the same blood samples, showing that similar results were obtained regardless of the screening method employed. This is important for the comparability of results from studies where these different screening methods have been used.

Limitations exist for any screening method for haemosporidians, whether using microscopy or molecular techniques. Birds with low parasitemia during the chronic phase of infection are always difficult to detect with microscopy creating the potential for misidentification of these birds as uninfected [27, 30]. Increasing the area of the blood film screened reduces the probability of false negative results [29], but adds considerable time to the screening process, 20 to 25 min per slide [29]. Even after adding additional screening time some infections will be missed. For example, a blood film from an individual with low parasitemia rarely contains all stages of haemosporidian development that are necessary for identification and/or adequate characterization of morphological species.

With molecular techniques, including nested PCR, low intensity infections can also be missed [29]. Molecular screening techniques based on PCR and Sanger sequencing also have lower ability to distinguish and identify mixed infections [61]. This is compounded by the fact that the host DNA is much more concentrated in samples than parasite DNA which somewhat affects the ability to detect haemosporidian DNA [62] or to PCR amplify larger fragments of parasite DNA, a necessity for the nested PCR protocol. This is evident in the results from this study, where only 77 % of the 693 samples identified as positive by real-time PCR were also identified as positive by nested PCR.

The goal of any new screening method is to provide an accurate estimate of parasite prevalence and to provide advantages over already established methods. The real-time PCR protocol proved as effective as the two most widely used molecular screening methods for haemosporidian parasites in birds [27, 35]. Although all three methods likely leave a small proportion of samples undetected, there are distinct advantages of the real-time protocol. The main advantage of this protocol is its ability to reliably and quickly detect haemosporidian infections. Since real-time PCR eliminates gel electrophoresis, the result for a full 96 or 384-well PCR plate are available in one hour (or sooner if fast running protocol and corresponding PCR mix is used). With the Fallon *et al*. [35] or Waldenström *et al*. [27] protocols not only is cycling time between 2.5 to 3.5 times longer respectively, there is also the added time of gel electrophoresis before results can be determined. Thus, the real-time protocol dramatically increases throughput of sample screening.

Of the three methods, only our real-time protocol uses a single reaction to screen for *Leucocytozoon* in addition to *Plasmodium* and *Haemoproteus* infections. The Fallon *et al*. [35] protocol was not designed to target *Leucocytozoon*. To amplify *Leucocytozoon* DNA with nested PCR a separate set of nested PCR amplifications are needed, the most widely used is the protocol of Hellgren *et al.* [26]. Inability to screen for all three genera in one nested PCR protocol increases the time and expense of screening for *Leucocytozoon* infections. This has led to a strong bias towards screening for *Haemoproteus* and *Plasmodium* only and ignoring *Leucocytozoon*, which explains why it is understudied. This is particularly true in areas of high host diversity, where the increased cost of PCR amplifications can make screening for *Leucocytozoon* prohibitive. Recent studies have shown that the *Leucocytozoon* diversity may be high in regions with high avian diversity [17] and in specific host populations [63]. Availability of a screening method that can amplify all three genera can aid in understanding the true diversity and ecology of all three genera of avian haemosporidian parasites. Until now, the only screening methods that could detect all three genera in a single procedure were microscopy and the restriction digestion protocol of Beadell & Fleischer [64], but both take significantly more time than the real-time PCR protocol and still require the use of nested PCR to amplify DNA for sequencing.

Although real-time PCR reagents are somewhat more expensive than those for standard PCR, it is more cost effective to use real-time PCR compared to the cost of running two to three rounds of regular/nested PCRs and associated gels for all samples. The cost advantage is even more evident when time and workforce cost are taken into consideration. This is especially beneficial when screening very large sets of hundreds or thousands of samples.

## Conclusions

Our real-time PCR assay proved as effective as two currently used molecular screening techniques, a single PCR screening assay [35] and nested PCR screening assays [26, 27]. However, the real-time protocol has the distinct advantage of detecting all three genera in a single reaction in at least half the time of these current methods. Therefore, throughput is significantly increased by greatly decreasing screening time and cost without loss of sensitivity. The ability to quickly and reliably screen avian blood samples is crucial for trying to understand the species richness and ecology of haemosporidian parasites, especially from highly diverse areas. The real-time protocol proposed here serves these purposes and provides a very useful tool in the expanding field of avian haemosporidian research.
